# Real-World Comparison of Biosimilar Ranibizumab (Ranieyes) and Innovator Ranibizumab (Lucentis/Accentrix) Across Multiple Retinal Vascular Diseases (The BRIO Study)

**DOI:** 10.3390/ph19050747

**Published:** 2026-05-11

**Authors:** Debdulal Chakraborty, Tushar Kanti Sinha, Sourav Sinha, Rupak Kanti Biswas, Arnab Das, Aniruddha Maiti, Ranabir Bhattacharya, Shouvick Dan, Dinesh Rungta, Shibashis Das

**Affiliations:** 1 Disha Eye Hospitals, Kolkata 700029, India; drtusharsinha@gmail.com (T.K.S.); drarnabdas_aiims@rediffmail.com (A.D.); ranabirbh@gmail.com (R.B.); shouvickdan@gmail.com (S.D.); drdineshrungta5@gmail.com (D.R.); 2 Nethralayam Superspeciality Eye Hospital, Kolkata 700073, India; ssinharetina@gmail.com (S.S.); rupakkbiswas@gmail.com (R.K.B.); 3 Global Eye Hospital, Kolkata 700054, India; write2maiti@gmail.com; 4 Sankara Jyoti Eye Institute, Kolkata 700060, India; drshibashisdas@gmail.com

**Keywords:** anti-VEGF, retinal vascular diseases, ranibizumab, biosimilar anti-VEGF, biosimilar ranibizumab

## Abstract

**Background:** Retinal vascular diseases, including neovascular age-related macular degeneration (nAMD), diabetic macular edema (DME), retinal vein occlusion (RVO), and myopic choroidal neovascularization (mCNV), often require repeated intravitreal anti-vascular endothelial growth factor (anti-VEGF) therapy. Although ranibizumab is well established, long-term affordability remains challenging. **Objective:** To compare the functional, anatomical, treatment-burden, and safety outcomes of biosimilar ranibizumab (Ranieyes) and innovator ranibizumab (Lucentis/Accentrix) in routine clinical practice. **Methods:** This multicenter retrospective comparative study included 4997 eyes from 3577 patients treated across five tertiary eye-care centers in India. The biosimilar group comprised 2543 eyes from 1812 patients (10,893 injections), and the innovator group comprised 2454 eyes from 1765 patients (10,136 injections). Eligible indications were nAMD, DME, BRVO, CRVO, mCNV, and an exploratory miscellaneous preoperative adjunct subgroup. BCVA (logMAR), central subfield thickness (CST; µm), injection burden, and ocular/systemic adverse events were assessed over 24 months. **Results:** Both groups showed early improvement in BCVA and CST across the major disease categories, followed by long-term stabilization. Between-group differences were generally small, not sustained over follow-up, and of limited clinical magnitude. Serious ocular and systemic adverse events were rare in both groups, and no new safety signal emerged. **Conclusions:** In this large real-world cohort, the biosimilar ranibizumab Ranieyes showed outcomes broadly comparable to innovator ranibizumab across the major retinal disease subgroups, although these findings should be interpreted as observational comparative evidence rather than formal proof of equivalence.

## 1. Introduction

Retinal vascular diseases—including neovascular age-related macular degeneration (nAMD), diabetic macular edema (DME), retinal vein occlusion (RVO), and myopic choroidal neovascularization (mCNV)—remain major causes of visual morbidity worldwide [1,2]. Their public-health impact continues to increase because of population aging, the growing prevalence of diabetes and hypertension, and the global rise in myopia [3,4,5]. These disorders are typically chronic and relapsing, and their long-term management imposes substantial clinical and financial burdens on patients and health systems.

Intravitreal anti-vascular endothelial growth factor (anti-VEGF) therapy has transformed the management of these conditions [6]. Ranibizumab, a humanized monoclonal antibody fragment targeting VEGF-A, has demonstrated efficacy across randomized clinical trials and real-world studies in nAMD, DME, RVO, and mCNV [7]. However, because many eyes require repeated injections over prolonged periods, cumulative treatment cost remains a major barrier to adherence, especially in low- and middle-income settings [8].

Biosimilars offer a potential solution to these access and affordability constraints [9]. By definition, a biosimilar is a biological product that is highly similar to a licensed reference product, with no clinically meaningful differences in quality, safety, or efficacy [9,10]. Their development relies on a stepwise comparability framework that includes analytical characterization, nonclinical evaluation, pharmacokinetic assessment, immunogenicity testing, and confirmatory clinical evidence [10,11].

Ranibizumab biosimilars are among the most studied ophthalmic biosimilars [9,10,11,12,13,14,15]. India was the first country to approve a ranibizumab biosimilar, and the subsequent introduction of additional biosimilar formulations has generated a substantial body of real-world evidence across retinal indications [11,12,13,14,15]. Several observational studies have reported visual, anatomical, and safety outcomes that are similar to those of innovator ranibizumab, without new safety concerns [12,13,14,15].

Despite this growing literature, important gaps remain. Much of the available evidence is disease-specific, non-comparative, or limited by relatively short follow-up. In addition, fewer multicenter real-world studies have evaluated a single ranibizumab biosimilar brand against innovator ranibizumab across heterogeneous retinal vascular diseases while simultaneously examining longitudinal outcomes, treatment burden, and safety. This gap provided the rationale for the present study.

In this context, we compared the clinical outcomes of biosimilar ranibizumab (Ranieyes; Lupin Pharmaceuticals, Mumbai, India) and innovator ranibizumab (Lucentis/Accentrix; Novartis, India) in a large multicenter real-world cohort. Our aim was to evaluate visual outcomes, anatomical response, injection burden, and safety across multiple retinal vascular diseases managed in routine practice.

## 2. Results

### 2.1. Study Population and Treatment Exposure

A total of 4997 eyes from 3577 patients were included: 2543 eyes from 1812 patients received biosimilar ranibizumab, and 2454 eyes from 1765 patients received innovator ranibizumab. Baseline demographic and study characteristics are summarized in Table 1. The mean age was 66.12 ± 14.13 years in the biosimilar group and 64.12 ± 11.25 years in the innovator group; male patients accounted for 63.7% and 56.5% of the two groups, respectively. There were 1210 treatment-naïve eyes in the biosimilar group and 1100 in the innovator group.

Disease distribution and total injection exposure are summarized in Table 2. Across the cohort, 10,893 injections were administered in the biosimilar group and 10,136 in the innovator group. Mean injection burden is shown in Table 3. Injection numbers were very similar in DME (4.45 ± 0.46 vs. 4.44 ± 0.49; *p* = 0.676), nAMD (5.96 ± 0.34 vs. 5.98 ± 0.41; *p* = 0.403), and CRVO (4.38 ± 0.12 vs. 4.35 ± 0.09; *p* = 0.055). Small absolute between-group differences were observed in BRVO (3.90 ± 0.08 vs. 3.97 ± 0.12; *p* < 0.001) and mCNV (1.82 ± 0.12 vs. 1.88 ± 0.15; *p* = 0.036). The miscellaneous subgroup also showed a difference in mean injections (1.50 ± 0.41 vs. 1.30 ± 0.22; *p* < 0.001), but this subgroup was exploratory and clinically heterogeneous.

### 2.2. Visual Outcomes

Visual outcomes by disease and treatment arm are summarized in Table 4 and Supplementary Table S1. Across all major disease categories, both groups showed early BCVA improvement followed by long-term stabilization. Between-group BCVA differences were generally small and not sustained over follow-up.

In DME, BCVA trajectories were closely overlapping throughout follow-up, with no between-group difference at baseline or any post-baseline visit. In nAMD, baseline BCVA was slightly worse in the innovator group, and a nominal between-group difference was observed at 12 months (0.42 ± 0.15 vs. 0.40 ± 0.15 logMAR; unadjusted *p* = 0.035), but the effect size was small, and the difference was not sustained at 18 or 24 months. In BRVO, baseline BCVA differed modestly, but post-treatment trajectories converged from 3 months onward. CRVO and mCNV also showed overlapping longitudinal BCVA profiles between treatment arms. Overall, the data support comparable visual trajectories between biosimilar and innovator ranibizumab, with isolated nominal differences that were small in magnitude and not persistent over time.

### 2.3. Anatomical Outcomes

Anatomical outcomes are summarized in Table 5 and Supplementary Table S2. Both treatment groups demonstrated marked CST reduction by 3 months in DME, nAMD, BRVO, CRVO, and mCNV, with maintenance of anatomical improvement through later follow-up.

Small between-group CST differences were observed at isolated early or mid follow-up visits, particularly in DME and nAMD, but these differences were not consistently sustained through 24 months and were not accompanied by a clinically meaningful separation in BCVA trajectories. Minor late CST fluctuations were observed in both arms, especially in DME and nAMD, but the magnitude of these changes was limited and likely reflects the variability inherent to real-world PRN treatment, recurrent disease activity, and measurement noise rather than a systematic difference between molecules.

**Figure 1:**
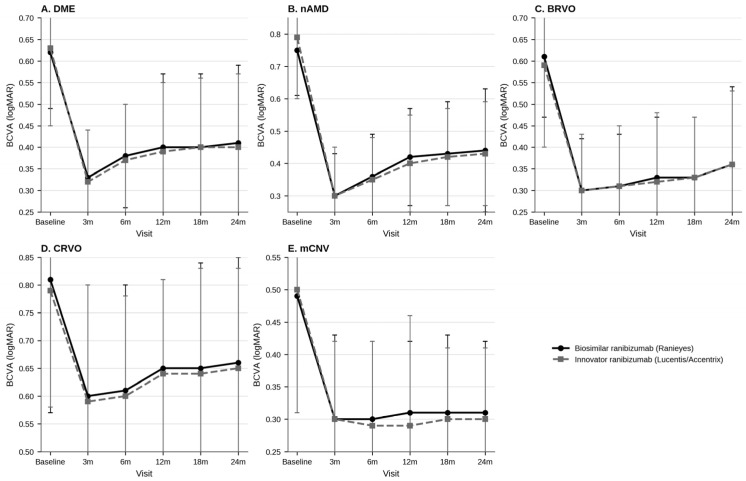
Longitudinal changes in best-corrected visual acuity (BCVA, logMAR) over 24 months in eyes with diabetic macular edema (DME), neovascular age-related macular degeneration (nAMD), branch retinal vein occlusion (BRVO), central retinal vein occlusion (CRVO), and myopic choroidal neovascularization (mCNV) treated with biosimilar ranibizumab or innovator ranibizumab. Data are presented as mean with standard deviation error bars. Lower logMAR values indicate better visual acuity. Between-group comparisons at each visit, including confidence intervals, effect sizes, and multiplicity-adjusted *p*-values, are provided in Supplementary Table S1.

**Figure 2:**
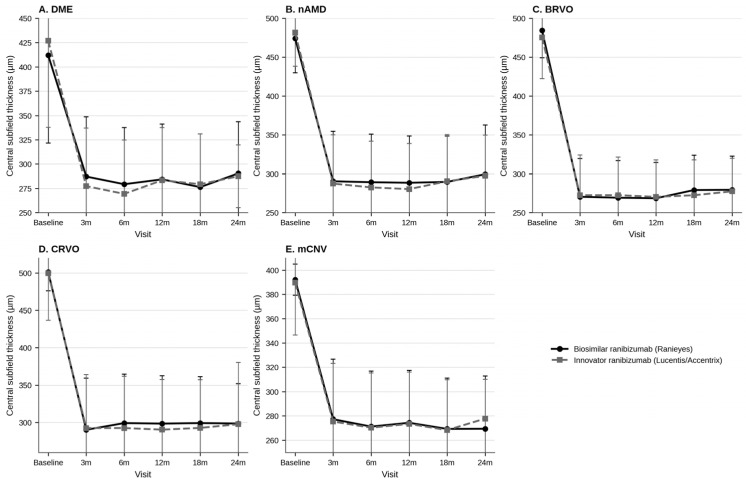
Longitudinal changes in central subfield thickness (CST, µm) over 24 months in eyes with diabetic macular edema (DME), neovascular age-related macular degeneration (nAMD), branch retinal vein occlusion (BRVO), central retinal vein occlusion (CRVO), and myopic choroidal neovascularization (mCNV) treated with biosimilar ranibizumab or innovator ranibizumab. Data are presented as mean with standard deviation error bars. Lower CST values indicate improvement in macular edema or exudative activity. Between-group comparisons at each visit, including confidence intervals, effect sizes, and multiplicity-adjusted *p*-values, are provided in Supplementary Table S2.

Taken together, the anatomical data indicate similar macular-thickness control with biosimilar and innovator ranibizumab over the study period. Disease-wise longitudinal trends in BCVA and CST are illustrated in Figure 1 and Figure 2, while detailed between-group statistical comparisons are provided in Supplementary Tables S1 and S2.

### 2.4. Safety Outcomes

Safety outcomes are summarized in Table 6 and Table 7. Most ocular adverse events were mild and injection-related, including ocular pain, watering, transient blurring, and subconjunctival hemorrhage. Serious ocular adverse events were rare. Endophthalmitis occurred in 1 of 10,893 injections (0.09/1000 injections) in the biosimilar group and 2 of 10,136 injections (0.20/1000 injections) in the innovator group (Fisher’s exact *p* = 0.612). Retinal pigment epithelial tears occurred in 21 and 24 eyes, respectively (*p* = 0.551).

Systemic adverse events were uncommon overall. Serious vascular events were infrequent, with myocardial infarction reported in 6 biosimilar-treated patients and 5 innovator-treated patients, and cerebrovascular accident in 3 and 5 patients, respectively; neither comparison was statistically significant. Because many non-serious adverse events were collected descriptively in routine practice, the normalized event rates in Table 6 and Table 7 should be interpreted cautiously.

Across more than 21,000 injections, no new ocular or systemic safety signal was identified for biosimilar ranibizumab.

## 3. Discussion

In this large multicenter real-world comparative study, the ranibizumab biosimilar Ranieyes showed visual, anatomical, and safety outcomes that were broadly comparable to those of innovator ranibizumab across DME, nAMD, BRVO, CRVO, and mCNV. The overall pattern was consistent across disease groups: early BCVA and CST improvement after treatment initiation, followed by maintenance with modest fluctuation over longer follow-up. However, the study was not designed as a formal equivalence or non-inferiority trial, and the findings should therefore be interpreted as comparative real-world evidence rather than proof of therapeutic equivalence.

The visual findings align with the expected disease-specific behavior of retinal vascular disorders in routine practice [16,17,18,19,20,21,22]. Eyes with CRVO showed more limited visual recovery than eyes with DME, BRVO, or mCNV, whereas eyes with nAMD and DME demonstrated early gains followed by relative stabilization, consistent with the chronic and relapse-prone nature of these conditions in real-world anti-VEGF care. The present study extends this literature by showing that these longitudinal patterns were similar for Ranieyes and innovator ranibizumab in routine multicenter practice.

The anatomical analysis similarly demonstrated early CST reduction in both groups. Although small numerical differences were seen at isolated visits, they were not sustained over time and were not mirrored by consistent BCVA divergence. In the nAMD subgroup, a nominal between-group BCVA difference was observed at 12 months; however, the absolute magnitude was small, the corresponding effect size was limited, and the difference was not sustained at 18 or 24 months. This pattern argues against a consistent clinically meaningful divergence between treatments while also underscoring that isolated time-point significance in a large retrospective dataset should be interpreted cautiously. Presenting effect sizes and 95% CIs therefore strengthens interpretation and directly addresses the need to go beyond statements of ‘no significant difference’.

Injection burden was also broadly similar between treatment groups, which reduces the likelihood that the overall outcome comparison was driven by major undertreatment in one arm. At the same time, the present study reflects real-world care, where retreatment patterns are shaped by affordability, logistics, adherence, and physician discretion. For this reason, small numerical differences in mean injections in BRVO or mCNV should be interpreted cautiously and in the clinical context rather than in isolation.

In the safety analysis, serious ocular complications such as endophthalmitis and severe inflammatory events were rare, and serious systemic vascular events were infrequent in both groups. These findings are in keeping with prior real-world evidence showing that ranibizumab biosimilars can achieve acceptable pharmacovigilance profiles when used in routine retinal practice [22,23,24,25,26,27,28,29,30,31,32,33].

The study also has practical implications for health systems. In settings where out-of-pocket expenditure remains an important determinant of treatment uptake and continuity, evidence supporting comparable real-world outcomes with a biosimilar agent may improve access to anti-VEGF therapy without materially compromising effectiveness or safety. At the participating hospitals during the study period, innovator ranibizumab was typically priced at approximately INR 25,000–30,000 ($270–325) per injection, whereas biosimilar ranibizumab was priced at approximately INR 12,000–16,000 ($129–173), indicating a substantial reduction in patient cost. This is consistent with prior biosimilar literature emphasizing affordability as a major driver of adoption and access [34,35]. Because patient-level economic data were not systematically collected, however, the present study should not be interpreted as a formal cost-effectiveness analysis.

Several limitations warrant emphasis. First, the retrospective design is inherently vulnerable to missing data, unmeasured confounding, and selection bias. Treatment allocation was influenced by affordability and patient preference rather than randomization; therefore, residual confounding cannot be excluded. Patients receiving innovator ranibizumab may have differed systematically from those receiving biosimilar ranibizumab in socioeconomic profile, access to care, adherence, and other measured or unmeasured clinical factors. No propensity score matching or multivariable adjustment was performed. Second, analyses were performed at the eye level, and some patients contributed both eyes; inter-eye correlation was therefore not fully modeled and may have influenced variance estimates. Third, the longitudinal analysis relied on transparent pointwise comparisons rather than reanalysis with more advanced repeated-measures or mixed-effects modeling, because raw-data reanalysis was not practical during the current revision; this should be considered when interpreting nominal time-point differences. Fourth, BCVA was derived from routine clinical Snellen measurements rather than standardized ETDRS assessments. Fifth, OCT measurements were obtained on three different devices across centers, although serial follow-up for a given patient was performed on the same device whenever feasible; accordingly, some inter-device variability may persist despite within-patient standardization. Sixth, follow-up and retreatment were determined in routine practice rather than by a protocolized trial schedule. Seventh, this study evaluated only one biosimilar brand (Ranieyes); therefore, the findings should not be generalized uncritically to all ranibizumab biosimilars. Finally, the miscellaneous subgroup was clinically heterogeneous and should be interpreted as exploratory. These limitations should be balanced against the strengths of the study, including its large sample size, multicenter design, inclusion of multiple retinal disease categories, and extensive injection-level safety experience.

## 4. Materials and Methods

### 4.1. Study Design and Setting

This retrospective, observational, multicenter comparative study was conducted at five tertiary eye-care centers in India between July 2022 and October 2025. The study adhered to the tenets of the Declaration of Helsinki and principles of Good Clinical Practice. Ethical approval was obtained from the respective institutional review boards and the central ethics committee (IRB Reg. No. ECR/846/Inst/WB/2016/RR-24). Written informed consent for participation in the study and use of anonymized clinical data was obtained from all patients.

### 4.2. Study Population

Medical records of consecutive patients receiving intravitreal innovator or biosimilar ranibizumab were reviewed. Eligible indications included nAMD, DME, BRVO, CRVO, mCNV, and a miscellaneous exploratory subgroup in which ranibizumab was used as a preoperative adjunct. The miscellaneous subgroup included eyes receiving anti-VEGF therapy before vitreoretinal surgery for tractional or non-clearing vitreous hemorrhage, proliferative diabetic retinopathy, neovascular glaucoma, or related indications. Although other ranibizumab biosimilars were used occasionally at participating centers during the study period, those eyes were few in number and were excluded to maintain a more homogeneous and interpretable comparison between one biosimilar formulation (Ranieyes) and the innovator molecule.

Inclusion criteria were age ≥ 18 years, a diagnosis established by clinical examination and multimodal imaging, treatment with either innovator or biosimilar ranibizumab as monotherapy, availability of baseline BCVA and OCT data, and follow-up to 24 months. Exclusion criteria were intraocular surgery other than uncomplicated cataract surgery or intravitreal therapy within 3 months before baseline, media opacity precluding reliable OCT acquisition, incomplete clinical/imaging records, and the presence of vitreomacular traction/adhesion or epiretinal membrane. The unit of analysis was the eye. If both eyes fulfilled eligibility criteria, both could be included; accordingly, some patients contributed bilateral eyes to the dataset.

### 4.3. Data Collection

Data were retrospectively extracted from the electronic medical record (EMR) systems of the participating centers using a predefined data-collection approach. Variables collected included age, sex, diagnosis, treatment-naïve status, baseline and follow-up BCVA, OCT-based CST measurements, treatment details including number of injections, and documented ocular and systemic adverse events. Systemic comorbidity variables and patient-level cost data were analyzed. Approximate contemporaneous patient prices at participating hospitals were used only for contextual interpretation in the Discussion.

### 4.4. Clinical and Imaging Assessment

BCVA was recorded using Snellen charts and converted to the logarithm of the minimum angle of resolution (logMAR) for analysis. CST was measured using spectral-domain OCT. Three OCT platforms were used across centers (Optovue OCT, Fremont, CA, USA; Cirrus 5000, Carl Zeiss Meditec; and Spectralis OCT, Heidelberg, Germany). For each patient, the same OCT platform was used for serial measurements whenever possible. CST was defined as the mean retinal thickness within the central 1 mm ETDRS subfield. Scans with segmentation errors or inadequate image quality were excluded.

### 4.5. Treatment Protocol

Treatment allocation was determined in routine practice after discussion with the patient regarding available options, cost, and the possible need for repeat injections. This affordability-driven allocation reflects real-world care but also represents a potential source of selection bias. Eyes with nAMD, DME, RVO, and mCNV were treated using a loading and/or pro re nata (PRN) strategy according to disease activity on clinical examination and OCT, with closer monthly reassessment during the initial phase. Reinjection during the first 6 months was generally advised for persistent or recurrent disease activity, including CST > 300 µm, together with BCVA of 20/40 or worse. In the miscellaneous subgroup, ranibizumab was used as a preoperative adjunct when deemed clinically beneficial.

### 4.6. Outcome Measures

The primary outcomes were change in BCVA (logMAR) and change in CST (µm) from baseline to follow-up among eyes with nAMD, DME, BRVO, CRVO, and mCNV. Secondary outcomes included injection burden, exploratory outcomes in the miscellaneous subgroup, and the incidence and nature of ocular and systemic adverse events.

### 4.7. Safety Assessment

Safety outcomes were obtained from the medical records. Ocular adverse events included intraocular inflammation, transient intraocular pressure elevation, vitreous hemorrhage, retinal pigment epithelial tears, endophthalmitis, and procedure-related events. Systemic adverse events included documented thromboembolic or other medically relevant events. Events were classified as serious or non-serious according to clinical severity and the need for intervention.

### 4.8. Statistical Analysis

Statistical analysis was performed using SPSS version 23 (IBM Corp., Armonk, NY, USA). Continuous variables are reported as mean ± standard deviation (SD), and categorical variables as counts and proportions. Distributional assumptions were checked using the Shapiro–Wilk test. Within-group changes over time were assessed using paired tests as appropriate in the original dataset. Between-group comparisons at individual time points were assessed using the independent-samples *t*-test (or Mann–Whitney U test when indicated), and categorical variables were compared using the chi-square test or Fisher’s exact test. For between-group contrasts, the mean difference with 95% confidence intervals (CIs) and Hedges g effect sizes are reported where feasible. Because repeated between-group comparisons were performed across multiple time points, adjusted *p*-values are provided in the Supplementary Tables using the Holm method. Analyses were therefore presented as transparent pointwise comparisons, and this should be considered when interpreting the findings. This study was not designed as a formal equivalence or non-inferiority trial; accordingly, the analyses should be interpreted as observational comparative assessments rather than proof of therapeutic equivalence. Analyses were performed at the eye level; because some patients contributed both eyes, inter-eye correlation may not have been fully accounted for, and this is acknowledged as a study limitation. The miscellaneous subgroup was considered exploratory. A two-sided *p*-value < 0.05 was considered statistically significant.

## 5. Conclusions

In conclusion, the ranibizumab biosimilar Ranieyes demonstrated real-world functional, anatomical, and safety outcomes that were broadly comparable to those of innovator ranibizumab across the major retinal disease subgroups. Disease-wise, trajectories in DME, BRVO, CRVO, and mCNV were closely overlapping between treatment arms, whereas nAMD showed an isolated nominal difference at 12 months that was small in magnitude and not sustained over longer follow-up. These findings should be interpreted as supportive observational comparative evidence rather than formal proof of equivalence. Because this study evaluated a single biosimilar brand, the results should not be generalized to all ranibizumab biosimilars. Further adjusted, disease-specific multicenter comparative studies across different biosimilar brands are warranted.

## Figures and Tables

**Table 1:** Baseline demographic and study characteristics.

Characteristic	Biosimilar	Innovator	*p*-Value
Patients, n	1812	1765	—
Eyes, n	2543	2454	—
Mean age, years	66.12 ± 14.13	64.12 ± 11.25	<0.001
Male sex, n (%)	1155 (63.7)	998 (56.5)	<0.001
Treatment-naïve eyes, n (%)	1210 (47.6)	1100 (44.8)	0.054
Total injections, n	10,893	10,136	—
DME eyes, n (%)	782 (30.8)	798 (32.5)	
nAMD eyes, n (%)	530 (20.8)	474 (19.3)	
BRVO eyes, n (%)	891 (35.0)	717 (29.2)	
CRVO eyes, n (%)	91 (3.6)	97 (4.0)	
mCNV eyes, n (%)	39 (1.5)	52 (2.1)	
Miscellaneous eyes, n (%)	210 (8.3)	316 (12.9)	<0.001 *

Note. Sex percentages are based on patient counts; disease-distribution and treatment-naïve percentages are based on eye counts. * Overall chi-square comparison across disease categories.

**Table 2:** Distribution of eyes and injections by indication.

Indication	Biosimilar: Eyes	Biosimilar: Injections	Innovator: Eyes	Innovator: Injections
DME	782	3480	798	3513
nAMD	530	3161	474	2840
BRVO	891	3477	717	2849
CRVO	91	399	97	423
mCNV	39	71	52	98
Miscellaneous	210	315	316	413
Total	2543	10,893	2454	10,136

Note. DME—diabetic macular edema, BRVO—branch retinal vein occlusion, mCNV = myopic choroidal neovascularization; nAMD = neovascular age-related macular degeneration.

**Table 3:** Mean number of injections over 1 year.

Diagnosis	Biosimilar (Mean ± SD)	Innovator (Mean ± SD)	Mean Difference (95% CI)	*p* Value	Hedges g
DME	4.45 ± 0.46	4.44 ± 0.49	0.01 (−0.04 to 0.06)	0.676	0.02
nAMD	5.96 ± 0.34	5.98 ± 0.41	−0.02 (−0.07 to 0.03)	0.403	−0.05
BRVO	3.90 ± 0.08	3.97 ± 0.12	−0.07 (−0.08 to −0.06)	<0.001	−0.70
CRVO	4.38 ± 0.12	4.35 ± 0.09	0.03 (−0.00 to 0.06)	0.055	0.28
mCNV	1.82 ± 0.12	1.88 ± 0.15	−0.06 (−0.12 to −0.00)	0.036	−0.43
Miscellaneous	1.50 ± 0.41	1.30 ± 0.22	0.20 (0.14 to 0.26)	<0.001	0.64

Note. Confidence intervals and Hedges g are based on between-group comparisons using the summary statistics available in the manuscript tables. The miscellaneous subgroup is exploratory.

**Table 4:** Best-corrected visual acuity (BCVA; logMAR) over 24 months.

Disease	Treatment	Baseline	3 Months	6 Months	12 Months	18 Months	24 Months
DME	Biosimilar	0.62 ± 0.13	0.33 ± 0.11	0.38 ± 0.12	0.40 ± 0.17	0.40 ± 0.17	0.41 ± 0.18
	Innovator	0.63 ± 0.18	0.32 ± 0.12	0.37 ± 0.13	0.39 ± 0.16	0.40 ± 0.16	0.40 ± 0.17
nAMD	Biosimilar	0.75 ± 0.14	0.30 ± 0.13	0.36 ± 0.13	0.42 ± 0.15	0.43 ± 0.16	0.44 ± 0.19
	Innovator	0.79 ± 0.19	0.30 ± 0.15	0.35 ± 0.13	0.40 ± 0.15	0.42 ± 0.15	0.43 ± 0.16
BRVO	Biosimilar	0.61 ± 0.14	0.30 ± 0.12	0.31 ± 0.12	0.33 ± 0.14	0.33 ± 0.14	0.36 ± 0.18
	Innovator	0.59 ± 0.19	0.30 ± 0.13	0.31 ± 0.14	0.32 ± 0.16	0.33 ± 0.14	0.36 ± 0.17
CRVO	Biosimilar	0.81 ± 0.24	0.60 ± 0.20	0.61 ± 0.19	0.65 ± 0.16	0.65 ± 0.19	0.66 ± 0.19
	Innovator	0.79 ± 0.21	0.59 ± 0.21	0.60 ± 0.18	0.64 ± 0.17	0.64 ± 0.19	0.65 ± 0.18
mCNV	Biosimilar	0.49 ± 0.18	0.30 ± 0.13	0.30 ± 0.12	0.31 ± 0.11	0.31 ± 0.12	0.31 ± 0.11
	Innovator	0.50 ± 0.19	0.30 ± 0.12	0.29 ± 0.13	0.29 ± 0.17	0.30 ± 0.11	0.30 ± 0.11

Note. Lower logMAR values indicate better visual acuity. Detailed between-group *p*-values, 95% confidence intervals, effect sizes, and Holm-adjusted *p*-values are provided in Supplementary Table S1. BCVA = best-corrected visual acuity; logMAR = logarithm of the minimum angle of resolution. DME—diabetic macular edema, BRVO—branch retinal vein occlusion, mCNV = myopic choroidal neovascularization; nAMD = neovascular age-related macular degeneration.

**Table 5:** Central subfield thickness (CST; µm) over 24 months.

Disease	Treatment	Baseline	3 Months	6 Months	12 Months	18 Months	24 Months
DME	Biosimilar	412.00 ± 90.29	287.23 ± 61.40	279.33 ± 58.20	284.33 ± 56.90	276.32 ± 54.80	290.43 ± 53.11
	Innovator	427.00 ± 89.19	277.34 ± 59.70	269.34 ± 55.30	283.42 ± 54.20	279.33 ± 51.60	287.45 ± 32.33
nAMD	Biosimilar	474.43 ± 44.50	290.43 ± 64.30	289.23 ± 61.70	288.53 ± 60.20	289.62 ± 58.90	299.43 ± 63.21
	Innovator	481.42 ± 43.10	287.44 ± 62.50	282.54 ± 59.40	280.32 ± 58.60	290.43 ± 60.10	297.45 ± 52.43
BRVO	Biosimilar	484.43 ± 35.30	270.43 ± 49.20	269.23 ± 47.50	268.53 ± 45.90	279.12 ± 44.80	279.43 ± 43.11
	Innovator	475.52 ± 53.10	272.41 ± 51.60	272.54 ± 48.90	270.32 ± 47.30	272.43 ± 45.20	277.45 ± 42.33
CRVO	Biosimilar	501.23 ± 25.30	290.23 ± 68.70	299.13 ± 65.40	298.43 ± 63.90	299.15 ± 61.80	298.53 ± 53.21
	Innovator	499.72 ± 63.11	292.51 ± 71.20	292.54 ± 68.90	290.42 ± 66.70	292.73 ± 64.10	297.65 ± 82.33
mCNV	Biosimilar	392.10 ± 12.90	277.23 ± 49.40	271.23 ± 45.60	274.33 ± 43.10	269.32 ± 41.70	269.43 ± 43.21
	Innovator	389.70 ± 43.19	275.34 ± 47.80	270.34 ± 44.90	273.42 ± 42.60	268.33 ± 41.20	277.75 ± 32.33

Note. Minor late fluctuations are visible in some subgroups but are not accompanied by sustained divergence in BCVA trajectories. Detailed between-group *p*-values, 95% confidence intervals, effect sizes, and Holm-adjusted *p*-values are provided in Supplementary Table S2. CST = central subfield thickness. DME—diabetic macular edema, BRVO—branch retinal vein occlusion, mCNV = myopic choroidal neovascularization; nAMD = neovascular age-related macular degeneration.

**Table 6:** Ocular adverse events.

Ocular Adverse Event	Biosimilar (n)	Rate/1000 Injections	Innovator (n)	Rate/1000 Injections	*p* Value
Ocular pain	620	56.92	579	57.12	0.953
Watering	332	30.48	350	34.53	0.102
Transient blurring	179	16.43	155	15.29	0.544
Subconjunctival hemorrhage	410	37.64	350	34.53	0.237
RPE tear	21	1.93	24	2.37	0.551
AC cells	11	1.01	9	0.89	0.826
Hypopyon	2	0.18	2	0.20	1.000
Endophthalmitis	1	0.09	2	0.20	0.612
Lens injury	2	0.18	3	0.30	0.677

Note. Rates are expressed per 1000 injections. *p*-values are descriptive between-group Fisher exact comparisons using total injections as the denominator. RPE = retinal pigment epithelium; IOP = intraocular pressure.

**Table 7:** Systemic adverse events.

Systemic Adverse Event	Biosimilar (n)	Rate/1000 Treated Eyes	Innovator (n)	Rate/1000 Treated Eyes	*p* Value
Raised blood pressure	110	43.26	78	31.78	0.037
Headache	78	30.67	66	26.89	0.447
Backache	69	27.13	56	22.82	0.365
Fall	12	4.72	14	5.70	0.696
Fracture of limbs	2	0.79	1	0.41	1.000
Myocardial infarction	6	2.36	5	2.04	1.000
Cerebrovascular accident	3	1.18	5	2.04	0.500

Note. Rates are expressed per 1000 treated eyes. Because non-serious systemic events were collected retrospectively from routine records, these rates should be interpreted descriptively. CVA = cerebrovascular accident; TIA = transient ischemic attack.

## Data Availability

The data presented in this study are available on request from the corresponding author. Data is not available publicly due to privacy restrictions.

## Supplementary Materials

The following supporting information can be downloaded at https://www.mdpi.com/article/10.3390/ph19050747/s1, Table S1. Between-group BCVA comparisons by disease and time point. Table S2. Between-group CST comparisons by disease and time point.
