# Supplementary material for: Real-World Comparison of Biosimilar Ranibizumab (Ranieyes) and Innovator Ranibizumab (Lucentis/Accentrix) Across Multiple Retinal Vascular Diseases (The BRIO Study)
Source: Pharmaceuticals (Basel). 2026 May 11;19(5):747. doi: 10.3390/ph19050747 (PMC13210171; doi:10.3390/ph19050747)
Supplement: Supplementary file 1 [file pharmaceuticals-19-00747-s001.zip › pharmaceuticals-4243452-supplementary.pdf]

Suggested Supplementary Statistical Tables

These supplementary tables are optional but strongly recommended because they directly address the reviewer's concerns regarding exact p values, confidence intervals, effect sizes, and multiple-comparison adjustment. They are derived from the summary statistics present in the submitted manuscript tables and should ideally be replaced or verified against the original patient-level analysis outputs before resubmission.

Supplementary Table S1. Between-group BCVA comparisons by disease and time point.

Disease	Time	Bio mean ± SD	Innovator mean ± SD	Mean diff (bio - inno)	95% CI	Hedges g	Unadjusted p	Holm-adjusted p
DME	Baseline	0.62 ± 0.13	0.63 ± 0.18	-0.01	-0.03 to 0.01	-0.06	0.205	0.820
DME	3 Months	0.33 ± 0.11	0.32 ± 0.12	0.01	-0.00 to 0.02	0.09	0.084	0.506
DME	6 Months	0.38 ± 0.12	0.37 ± 0.13	0.01	-0.00 to 0.02	0.08	0.112	0.561
DME	12 Months	0.40 ± 0.17	0.39 ± 0.16	0.01	-0.01 to 0.03	0.06	0.229	0.820
DME	18 Months	0.40 ± 0.17	0.40 ± 0.16	0.00	-0.02 to 0.02	0.00	1.000	1.000
DME	24 Months	0.41 ± 0.18	0.40 ± 0.17	0.01	-0.01 to 0.03	0.06	0.257	0.820
Wet AMD	Baseline	0.75 ± 0.14	0.79 ± 0.19	-0.04	-0.06 to -0.02	-0.24	<0.001	0.001
Wet AMD	3 Months	0.30 ± 0.13	0.30 ± 0.15	0.00	-0.02 to 0.02	0.00	1.000	1.000
Wet AMD	6 Months	0.36 ± 0.13	0.35 ± 0.13	0.01	-0.01 to 0.03	0.08	0.224	0.896
Wet AMD	12 Months	0.42 ± 0.15	0.40 ± 0.15	0.02	0.00 to 0.04	0.13	0.035	0.176
Wet AMD	18 Months	0.43 ± 0.16	0.42 ± 0.15	0.01	-0.01 to 0.03	0.06	0.307	0.921
Wet AMD	24 Months	0.44 ± 0.19	0.43 ± 0.16	0.01	-0.01 to 0.03	0.06	0.366	0.921
BRVO	Baseline	0.61 ± 0.14	0.59 ± 0.19	0.02	0.00 to 0.04	0.12	0.019	0.113
BRVO	3 Months	0.30 ± 0.12	0.30 ± 0.13	0.00	-0.01 to 0.01	0.00	1.000	1.000

BRVO	6 Months	0.31 ± 0.12	0.31 ± 0.14	0.00	-0.01 to 0.01	0.00	1.000	1.000
BRVO	12 Months	0.33 ± 0.14	0.32 ± 0.16	0.01	-0.00 to 0.02	0.07	0.188	0.941
BRVO	18 Months	0.33 ± 0.14	0.33 ± 0.14	0.00	-0.01 to 0.01	0.00	1.000	1.000
BRVO	24 Months	0.36 ± 0.18	0.36 ± 0.17	0.00	-0.02 to 0.02	0.00	1.000	1.000
CRVO	Baseline	0.81 ± 0.24	0.79 ± 0.21	0.02	-0.05 to 0.09	0.09	0.545	1.000
CRVO	3 Months	0.60 ± 0.20	0.59 ± 0.21	0.01	-0.05 to 0.07	0.05	0.738	1.000
CRVO	6 Months	0.61 ± 0.19	0.60 ± 0.18	0.01	-0.04 to 0.06	0.05	0.712	1.000
CRVO	12 Months	0.65 ± 0.16	0.64 ± 0.17	0.01	-0.04 to 0.06	0.06	0.678	1.000
CRVO	18 Months	0.65 ± 0.19	0.64 ± 0.19	0.01	-0.04 to 0.06	0.05	0.719	1.000
CRVO	24 Months	0.66 ± 0.19	0.65 ± 0.18	0.01	-0.04 to 0.06	0.05	0.712	1.000
Myopic CNVM	Baseline	0.49 ± 0.18	0.50 ± 0.19	-0.01	-0.09 to 0.07	-0.05	0.799	1.000
Myopic CNVM	3 Months	0.30 ± 0.13	0.30 ± 0.12	0.00	-0.05 to 0.05	0.00	1.000	1.000
Myopic CNVM	6 Months	0.30 ± 0.12	0.29 ± 0.13	0.01	-0.04 to 0.06	0.08	0.705	1.000
Myopic CNVM	12 Months	0.31 ± 0.11	0.29 ± 0.17	0.02	-0.04 to 0.08	0.13	0.499	1.000
Myopic CNVM	18 Months	0.31 ± 0.12	0.30 ± 0.11	0.01	-0.04 to 0.06	0.09	0.685	1.000
,								
Myopic CNVM	24 Months	0.31 ± 0.11	0.30 ± 0.11	0.01	-0.04 to 0.06	0.09	0.669	1.000

Note. Holm-adjusted p values were calculated within each disease across the six repeated time-point comparisons. BCVA = best-corrected visual acuity; logMAR = logarithm of the minimum angle of resolution.

Supplementary Table S2. Between-group CST comparisons by disease and time point.

Disease	Time	Bio mean ± SD	Innovator mean ± SD	Mean diff (bio - inno)	95% CI	Hedges g	Unadjusted p	Holm-adjusted p
DME	Baseline	412.00 ± 90.29	427.00 ± 89.19	-15.00	-23.86 to -6.14	-0.17	<0.001	0.005
DME	3 Months	287.23 ± 61.40	277.34 ± 59.70	9.89	3.91 to 15.87	0.16	0.001	0.005
DME	6 Months	279.33 ± 58.20	269.34 ± 55.30	9.99	4.39 to 15.59	0.18	<0.001	0.003
DME	12 Months	284.33 ± 56.90	283.42 ± 54.20	0.91	-4.58 to 6.40	0.02	0.745	0.745
DME	18 Months	276.32 ± 54.80	279.33 ± 51.60	-3.01	-8.26 to 2.24	-0.06	0.261	0.538
DME	24 Months	290.43 ± 53.11	287.45 ± 32.33	2.98	-1.37 to 7.33	0.07	0.179	0.538
Wet AMD	Baseline	474.43 ± 44.50	481.42 ± 43.10	-6.99	-12.42 to -1.56	-0.16	0.012	0.070
Wet AMD	3 Months	290.43 ± 64.30	287.44 ± 62.50	2.99	-4.87 to 10.85	0.05	0.456	1.000
Wet AMD	6 Months	289.23 ± 61.70	282.54 ± 59.40	6.69	-0.81 to 14.19	0.11	0.081	0.322
Wet AMD	12 Months	288.53 ± 60.20	280.32 ± 58.60	8.21	0.85 to 15.57	0.14	0.029	0.145
Wet AMD	18 Months	289.62 ± 58.90	290.43 ± 60.10	-0.81	-8.20 to 6.58	-0.01	0.830	1.000
Wet AMD	24 Months	299.43 ± 63.21	297.45 ± 52.43	1.98	-5.19 to 9.15	0.03	0.588	1.000
BRVO	Baseline	484.43 ± 35.30	475.52 ± 53.10	8.91	4.38 to 13.44	0.20	<0.001	<0.001
BRVO	3 Months	270.43 ± 49.20	272.41 ± 51.60	-1.98	-6.95 to 2.99	-0.04	0.435	1.000
BRVO	6 Months	269.23 ± 47.50	272.54 ± 48.90	-3.31	-8.06 to 1.44	-0.07	0.172	0.688
BRVO	12 Months	268.53 ± 45.90	270.32 ± 47.30	-1.79	-6.38 to 2.80	-0.04	0.445	1.000
BRVO	18 Months	279.12 ± 44.80	272.43 ± 45.20	6.69	2.26 to 11.12	0.15	0.003	0.016
BRVO	24 Months	279.43 ± 43.11	277.45 ± 42.33	1.98	-2.22 to 6.18	0.05	0.355	1.000

CRVO	Baseline	501.23 ± 25.30	499.72 ± 63.11	1.51	-12.21 to 15.23	0.03	0.828	1.000
CRVO	3 Months	290.23 ± 68.70	292.51 ± 71.20	-2.28	-22.41 to 17.85	-0.03	0.823	1.000
CRVO	6 Months	299.13 ± 65.40	292.54 ± 68.90	6.59	-12.73 to 25.91	0.10	0.502	1.000
CRVO	12 Months	298.43 ± 63.90	290.42 ± 66.70	8.01	-10.78 to 26.80	0.12	0.401	1.000
CRVO	18 Months	299.15 ± 61.80	292.73 ± 64.10	6.42	-11.70 to 24.54	0.10	0.485	1.000
CRVO	24 Months	298.53 ± 53.21	297.65 ± 82.33	0.88	-18.96 to 20.72	0.01	0.930	1.000
Myopic CNVM	Baseline	392.10 ± 12.90	389.70 ± 43.19	2.40	-10.26 to 15.06	0.07	0.706	1.000
Myopic CNVM	3 Months	277.23 ± 49.40	275.34 ± 47.80	1.89	-18.65 to 22.43	0.04	0.855	1.000
Myopic CNVM	6 Months	271.23 ± 45.60	270.34 ± 44.90	0.89	-18.20 to 19.98	0.02	0.926	1.000
Myopic CNVM	12 Months	274.33 ± 43.10	273.42 ± 42.60	0.91	-17.16 to 18.98	0.02	0.920	1.000
Myopic CNVM	18 Months	269.32 ± 41.70	268.33 ± 41.20	0.99	-16.49 to 18.47	0.02	0.911	1.000
Myopic CNVM	24 Months	269.43 ± 43.21	277.75 ± 32.33	-8.32	-24.77 to 8.13	-0.22	0.317	1.000

Note. Holm-adjusted p values were calculated within each disease across the six repeated time-point comparisons. CST = central subfield thickness; mCNV = myopic choroidal neovascularization; nAMD = neovascular age-related macular degeneration.
